# Reliability Modeling of Wind Turbine Gearbox System Considering Failure Correlation Under Shock–Degradation

**DOI:** 10.3390/s25144425

**Published:** 2025-07-16

**Authors:** Xiaojun Liu, Ziwen Wu, Yiping Yuan, Wenlei Sun, Jianxiong Gao

**Affiliations:** School of Mechanical Engineering, Xinjiang University, Urumqi 830046, China

**Keywords:** wind turbine gearbox, shock–degradation coupling, mixed Copula, reliability modeling, failure correlation

## Abstract

To address traditional methods’ limitations in neglecting the interaction between random shock loads and progressive degradation, as well as failure correlations, this study proposes a dynamic reliability framework integrating Gamma processes, homogeneous Poisson processes (HPP), and mixed Copula functions. The framework develops a wind turbine gearbox reliability model under shock–degradation coupling while quantifying failure correlations. Gamma processes characterize continuous degradation, with parameters estimated from P-S-N curves. Based on stress–strength interference theory, random shocks within damage thresholds are integrated to form a coupled reliability model. A Gumbel–Clayton–Frank mixed Copula with a multi-layer nested algorithm quantifies failure correlations, with correlation parameters estimated via the RSS principle and genetic algorithms. Validation using a 2 MW gearbox’s planetary gear-stage system covers four scenarios: natural degradation, shock–degradation coupling, and both scenarios with failure correlations. The results show that compared to independent assumptions, the model accelerates reliability decline, increasing failure rates by >37%. Relative to natural degradation-only models, failure rates rise by >60%, validating the model’s effectiveness and alignment with real-world operational conditions.

## 1. Introduction

With the upscaling deployment of wind turbine units and progressive growth in single-unit capacity, transmission systems now confront three predominant challenges: high-cycle loading, non-stationary operating conditions, and random shock loads. These combined stressors have been exacerbated to critical levels in contemporary designs. As the core transmission component in wind turbine generators, gearboxes endure combined impacts from shock loads and progressive degradation during prolonged operation. This results in dynamic coupling effects among multimodal failure mechanisms including tooth surface wear, tooth root bending fatigue, and bearing fatigue, leading to significant time-varying reliability attenuation characteristics [1,2,3]. Therefore, developing reliable gearbox evolution models that account for dynamic load transfer mechanisms and failure interdependencies is critical for ensuring the operational integrity and power generation stability of modern wind turbines.

Contemporary investigations on wind turbine gearbox reliability have predominantly concentrated on three principal research streams: dynamic loading characterization, failure interdependency assessment, and data-driven prognostic approaches; these methodological frameworks constitute the current paradigm in the wind energy sector. (1) In the domain of dynamic loading characterization, Qin et al. [4] formulated the equivalent load cumulative distribution function through stochastic process theory integrated with stress–strength interference theory. Through second-moment perturbation analysis, their work elucidated the time-dependent reliability evolution mechanism in gear transmission systems. An et al. [5] implemented Markovian state transition modeling to predict gear strength degradation trajectories, subsequently developing a prognostic assessment framework for the dynamic reliability envelope of wind turbine gearbox systems. Nejad et al. [6] conducted a systematic investigation into the progressive fatigue damage accumulation in gear systems subjected to stochastic wind loading, developing a damage evolution framework through the lens of Miner’s linear cumulative damage theory. Wang et al. [7] developed a dynamic diagnostic framework for wind turbine gearboxes by synergistically integrating enhanced wavelet packet decomposition with heterogeneous ensemble learning architectures, enabling precise condition monitoring and fault identification through operational vibration signature analysis. Li et al. [8] incorporated time-varying meshing stiffness and bearing clearance effects into their dynamic simulation framework, establishing a high-fidelity numerical model for wind turbine gearbox vibration analysis under transient operational conditions. Sun et al. [9] developed a dynamic modeling methodology for wind turbine gearboxes by integrating finite element analysis with multi-body system dynamics. (2) Within the field of failure correlation analysis, Ditlevsen [10] introduced the foundational framework of failure mode correlation, establishing critical theoretical support for streamlined reliability evaluation in mechanical systems. Yang Tengfei [11] established the degree of failure correlation between gearbox components through developing a joint distribution model integrating strength degradation with failure correlation. Deng Zhiming [12] investigated the degradation–failure correlation in high-speed shaft gears via Copula functions, establishing a reliability model for wind turbine gearboxes that accounts for coupled effects of multiple failure modes. Chen Wenhua et al. [13] investigated the fatigue performance of gears, developed two Copula-based fatigue reliability models, and conducted fatigue reliability assessment through quantitative analysis. Bao Zhaowei [14] developed a three-stage systematic optimization framework through the incorporation of Copula functions to address interdependencies between multiple performance parameters. (3) To address data scarcity, Li et al. [15] developed a performance degradation-based hidden Markov model, enabling reliability evaluation of wind turbine gearboxes under small-sample conditions. Zhang et al. [16] investigated the application of small-sample learning in the fault diagnosis of wind turbine gearboxes. Li et al. [17] postulated that expert knowledge serves as a critical supplementary source under data scarcity conditions, where domain-specific expertise can be formalized into quantifiable reliability models through methodologies such as fuzzy logic.

However, current studies exhibit the following critical limitations: Firstly, existing models predominantly concentrate on natural degradation processes (e.g., Gamma process [18]), yet they neglect to incorporate the nonlinear accelerating effects of random shocks on material residual strength degradation, leading to the inadequate characterization of shock–degradation coupled mechanisms. Secondly, conventional approaches predominantly rely on either independent failure mode assumptions [19] or employ single Copula functions (e.g., Gumbel or Clayton Copula) for correlation characterization [20], which significantly impedes the accurate modeling of multivariate tail dependence characteristics in multi-failure-mode systems. Thirdly, models employing independent assumptions or linear cumulative damage theory [6] demonstrate considerable prediction inaccuracies when subjected to coupled dynamic loading and degradation interactions [4], while also lacking computationally efficient algorithms for resolving multi-scale failure correlations.

In summary, this study develops a dynamic reliability modeling framework integrating Gamma processes, homogeneous Poisson processes (HPP), and mixed Copula functions, establishing a wind turbine gearbox reliability model that explicitly addresses failure correlation under coupled shock–degradation mechanisms. The principal components of this study are as follows: (1) A reliability model incorporating shock–degradation coupling mechanisms was developed for wind turbine gearbox systems. The continuous cumulative degradation process was modeled using Gamma processes, followed by formulation of the reliability framework through stress–strength interference theory. Parameter estimation was subsequently achieved via P-S-N curve analysis. (2) A Gumbel–Clayton–Frank mixed Copula function was implemented to characterize multi-scale failure correlations, with a hierarchical nested algorithm developed for modeling interdependencies. This framework enabled construction of a wind turbine gearbox system reliability model addressing failure correlation mechanisms under coupled shock–degradation interactions. (3) Theoretical validation: Employing a 2 MW wind turbine planetary gear system as the validation scenario, the proposed modeling methodology was numerically validated for both technical feasibility and theoretical consistency.

## 2. Reliability Modeling with Shock–Degradation Coupling Mechanisms

**Figure 1:**
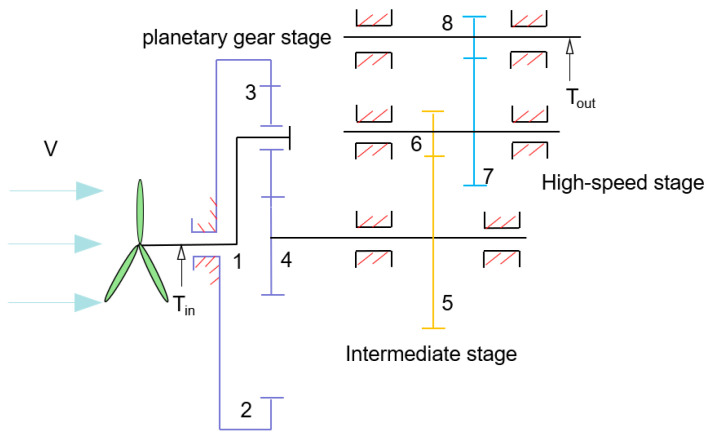
Schematic configuration of wind turbine gearbox transmission system. V: wind velocity; T_in_: input torque; 1: planet carrier; 2: internal gear; 3: planetary gear; 4: sun gear; 5–8: helical gears; T_out_: output torque.

The gearbox functions as a pivotal transmission component in wind turbines, mechanically linking the rotor with the generator. By employing multi-stage gear meshing, this system amplifies rotational speed to transform the rotor’s low-speed, high-torque input into generator-compatible high-speed, low-torque output, thereby enabling efficient wind-to-electrical energy conversion. This study employs a 2 MW doubly fed induction wind turbine generator system as the research subject. The gearbox configuration, illustrated in Figure 1, features a planetary-parallel shaft transmission arrangement comprising a planetary stage collaboratively integrated with two parallel-shaft helical gear stages. The low-speed, high-torque input T_in_ from the rotor actuates planetary gear 3 to orbit the sun gear 4 through the planet carrier 1. Leveraging the inherent high transmission ratio of planetary gearing, this stage accomplishes primary speed reduction while enabling optimized load distribution across meshing components. The sun gear output shaft channels decelerated torque to the intermediate-stage parallel-axis helical gears 5 and 6. Through helical tooth engagement, this arrangement achieves secondary speed amplification while enabling enhanced torque distribution uniformity across the transmission system. The high-speed stage helical gears 7 and 8 amplify rotational velocity to yield a high-speed, low-torque output T_out_ compatible with generator input requirements.

The primary failure modes of wind turbine gearboxes, resulting from the combined effects of long-term alternating load fatigue accumulation and coupled dynamic interactions with impact loads under extreme operating conditions, are primarily manifested in gear components. These include contact fatigue, surface micropitting, and bending fatigue fractures at the tooth root [21]. From the perspective of failure mechanisms, cyclic loading induces microcrack propagation through stress cycling effects, leading to contact fatigue pitting degradation. Conversely, shock loads generate transient stress concentrations that precipitate catastrophic failures such as bending-induced tooth fracture through instantaneous overload conditions. The progressive degradation induced by cyclic loading progressively diminishes the system’s load-bearing capacity, thereby inducing overload conditions where previously sustainable shock loads exceed the material’s ultimate strength threshold. Concurrently, the cumulative degradation process initiates a progressive decline in the shock resistance capacity of the gearbox system, establishing a mutually reinforcing failure mechanism [22]. Therefore, the two failure modes exhibit reciprocal interactions that exacerbate system degradation.

**Figure 2:**
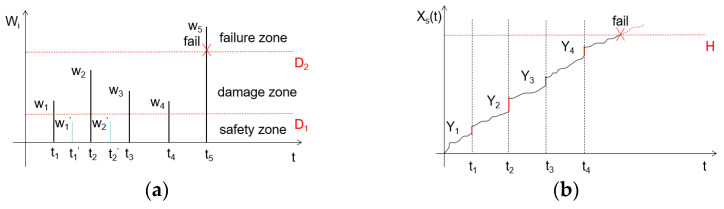
Degradation mechanisms in wind turbine gearbox systems under coupled shock–degradation loading conditions: (**a**) failure progression in wind turbine gearbox systems under random shock loading; (**b**) degradation progression in wind turbine gearbox systems under random shock loading.

To ensure analytical tractability while maintaining fidelity to wind turbine operational boundary conditions, the following assumptions are established for the gearbox shock–degradation coupling system model:

(1) As illustrated in Figure 2a, the system’s shock response can be categorized into three distinct operational regimes based on the shock load intensity $W_{i}$: (i) the safety zone $\left(W_{i}\leq D_{1}\right)$, (ii) the damage zone ($D_{1}<W_{i}<D_{2}$), and (iii) the failure zone $\left(W_{i}\geq D_{2}\right)$. The amplitude of individual shock loads follows an independent normal distribution, while the occurrence of random shocks adheres to a Poisson process characterized by time-dependent intensity λ(*t*).

In accordance with Poisson process theory, the interarrival times of random shock loads in wind turbine gearbox systems follow an exponential distribution with parameter λ, as depicted in Figure 2a. The shock intensity sequence $\left\{W_{i}\right\}$ is assumed to comprise independent and identically distributed random variables following a normal distribution, denoted as $W_{i}∼N(\mu_{W},\sigma_{W}^{2})$.

(2) As depicted in Figure 2, wind turbine gearbox systems exhibit two distinct failure mechanisms during operational service: progressive degradation and catastrophic failure. As illustrated in Figure 2b, random shock loads accelerate the degradation process in wind turbine gearbox systems. The total system degradation $X_{s}(t)$ at time t constitutes a superposition of inherent degradation $X\left(t\right)$ and shock-induced cumulative degradation $S\left(t\right)$. System failure occurs when $X_{s}(t)$ attains the predefined failure threshold H.

**Figure 3:**
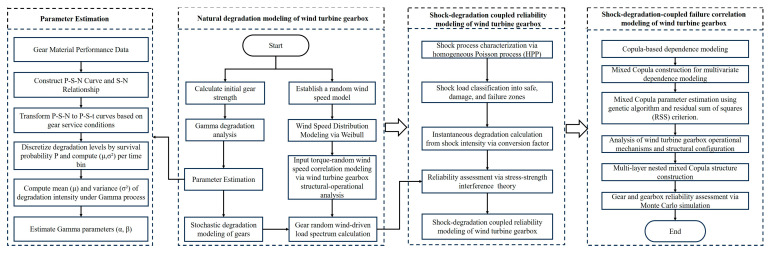
Flowchart for coupled shock–degradation reliability modeling of wind turbine gearbox.

Building upon the aforementioned modeling assumptions, a comprehensive reliability model for wind turbine gearbox systems incorporating shock–degradation coupling mechanisms has been developed. The model development process is presented in Figure 3.

### 2.1. Natural Degradation Modeling of Wind Turbine Gearbox Systems Using Gamma Process

In the field of reliability engineering, the gamma process-based degradation model has gained widespread adoption due to its ability to accurately capture non-negative monotonic degradation processes while maintaining well-defined probabilistic properties. The gamma process is a non-negative, monotonically increasing, continuous-time stochastic process with independent and stationary increments, widely employed to model the continuous cumulative degradation of mechanical systems. Therefore, the gamma process is employed to model the natural degradation of wind turbine gearbox systems.

Let $X\left(t\right)$ represent the cumulative degradation at time t. The probability density function $f_{X}(x)$ of the gearbox system is governed by the gamma process parameters and its stochastic properties:

(1) $$f_{X}\left(x|\alpha \left(t\right),\beta \right)=\frac{1}{\Gamma \left(\alpha \right)}\beta^{\alpha \left(t\right)}x^{\alpha \left(t\right)-1}e^{-\beta x}I_{\left(0,\infty \right)}\left(x\right)$$

where $\Gamma (\alpha )=\int_{0}^{\infty }x^{\alpha (t)-1}e^{-x}dx$ is gamma function. x∈(0,∞), $I_{\left(0,\infty \right)}\left(x\right)=1$, x∉(0,∞), $I_{\left(0,\infty \right)}\left(x\right)=0$; α(t) is the time-dependent shape parameter $\left(\alpha (t)>0\right)$, and β is the scale parameter $\left(\beta >0\right)$.

To quantitatively characterize both the mean degradation trend and stochastic behavior of wind turbine gearbox system degradation $X\left(t\right)$, the gamma process statistical properties are derived as follows:

Mean degradation function:

(2) $$E\left(X\left(t\right)\right)=\frac{\alpha \left(t\right)}{\beta }$$

Variance degradation function:

(3) $$D\left(X\left(t\right)\right)=\frac{\alpha \left(t\right)}{\beta^{2}}$$

Given the prescribed failure threshold H, the reliability function $R\left(t\right)$ of wind turbine gearbox systems at time t is defined as the probability that the cumulative degradation remains below H:

(4) $$R\left(t\right)=P\left\{X\left(t\right)<H\right\}=\int_{0}^{H}f\left(x\right)dx=\frac{\int_{0}^{H\beta }u^{\alpha \left(t\right)-1}e^{-\beta u}du}{\Gamma \left(\alpha \left(t\right)\right)}$$

The shape parameter α(t) and scale parameter β of the natural degradation process are estimated through the parameter estimation method outlined in Modeling Flowchart 3.

### 2.2. Reliability Modeling of Wind Turbine Gearbox Systems with Shock–Degradation Coupling

During operational service, wind turbine gearbox systems are subjected to random shock loads. Assuming the shock arrival events follow a homogeneous Poisson process with parameter λ, let $N\left(t\right)$ denote the total number of shocks occurring within the time interval [0,t]. For any subsequent interval [s,s+t], the impact count n conforms to the corresponding probabilistic characteristics:

(5) $$P\left\{N\left(s+t\right)-N\left(s\right)=n\right\}=\frac{e^{-\lambda t}{\left(\lambda t\right)}^{n}}{n!}\;(n=0,1,2,\cdots )$$

Given that subcritical shock loads within the operational safety zone exert a negligible influence on wind turbine gearbox system degradation, the defined load intensity thresholds $W_{1}^{\prime }$ and $W_{2}^{\prime }$ (illustrated in Figure 2a) are excluded from the model formulation.

Define $D=D_{2}$ as the critical impact intensity threshold inducing sudden failure. The probability of shock loads $W_{i}$ remaining below D within the subcritical damage zone is quantified as follows:

(6) $$p_{1}=P\left\{W_{i}<D\right\}=\Phi \left(\frac{D-\mu_{W}}{\sigma_{W}}\right)$$

Based on the Poisson process decomposition theorem [23], the shock arrival rate inducing cumulative damage-induced failure follows a Poisson distribution with parameter ${\lambda p}_{1}$. Let $Y_{i}$ denote the instantaneous degradation increment of the gearbox system caused by the shock strength $W_{i}(W_{i}<D)$:

(7) $$Y_{i}=\kappa W_{i}$$

where κ is the scaling factor. For $W_{i}∼N(\mu_{W},\sigma_{W}^{2})$; applying the linear transformation properties of normal distributions, it follows that $Y_{i}∼N(\kappa \mu_{W},{\kappa \sigma }_{W}^{2})$.

Let $N_{1}\left(t\right)$ denote the number of shock arrivals within the subcritical damage zone. Consequently, the total cumulative degradation of the gearbox system induced by shock loads $\left\{W_{i}\right\}$ over the time interval [0,t] is

(8) $$S\left(t\right)=\sum_{i=1}^{N_{1}\left(t\right)}Y_{i},N_{1}\left(t\right)>0$$

If $N_{1}\left(t\right)=n$, the probability density function of the cumulative degradation $S\left(t\right)$ for the gearbox system is

(9) $$F_{S}=\sum_{i=1}^{n}Y_{i}=\Phi \left(\frac{s-n\kappa \mu_{W}}{\kappa \sigma_{W}\sqrt{n}}\right)$$

In summary, considering the coupled shock–degradation failure mechanism, the cumulative degradation of wind turbine gearbox systems is

(10) $$X_{S}\left(t\right)=X\left(t\right)+S\left(t\right)=X\left(t\right)+\sum_{i=1}^{N_{1}\left(t\right)}Y_{i}$$

Based on the gamma process degradation model, integrating the effects of shock-induced degradation while excluding catastrophic failure events ($W_{i}<D$), the reliability of wind turbine gearbox systems under coupled shock–degradation mechanisms is formulated as

(11) $$\begin{array}{cc} R_{X}\left(t\right) & =P\left(X_{S}\left(t\right)<H\right)\\ & =P\left(X\left(t\right)+S\left(t\right)<H\right)\\ & =\sum_{n=0}^{\infty }P\left(X\left(t\right)+S\left(t\right)<H|N_{1}\left(t\right)=n\right)P\left(N_{1}\left(t\right)=n\right)\;\\ & =P\left(X\left(t\right)<H|N_{1}\left(t\right)=0\right)P\left(N_{1}\left(t\right)=0\right)\\ & \;\;\;+\sum_{n=1}^{\infty }P(X\left(t\right)+\sum_{i=1}^{n}Y_{i}<H|N_{1}t=n)P\left(N_{1}\left(t\right)=n\right)\;\\ & =P\left(X\left(t\right)<H\right)e^{-\lambda P_{1}t}+\;\sum_{n=1}^{\infty }P\left(X\left(t\right)<H-\sum_{i=1}^{n}Y_{i}\;\right)\frac{{\left(\lambda P_{1}t\right)}^{n}e^{-\lambda P_{1}t}}{n!}\\ & =\frac{\int_{0}^{H\beta }u^{\alpha \left(t\right)-1}e^{-\beta u}du\;}{\Gamma \left(\alpha \left(t\right)\right)}e^{-\lambda P_{1}t}+\sum_{n=1}^{\infty }\;\frac{\int_{0}^{\left(H-\sum_{i=1}^{n}Y_{i}\;\right)\beta }u^{\alpha \left(t\right)-1}e^{-\beta u}du\;}{\Gamma \left(\alpha \left(t\right)\right)}\frac{{\left(\lambda P_{1}t\right)}^{n}e^{-\lambda P_{1}t}}{n!} \end{array}$$

### 2.3. Failure-Correlated Reliability Modeling of Wind Turbine Gearbox Systems Under Shock–Degradation Coupling

Wind turbine gearbox systems constitute a multi-component, multi-failure-mode coupled system. To enhance the fidelity of the reliability model under shock–degradation coupling to actual operational conditions, failure correlations are analyzed across three hierarchical levels: failure mechanisms, component interactions, and system-wide dynamics.

#### 2.3.1. Failure-Correlation Quantification Methodology

To characterize failure correlation in mechanical systems, the Copula function—initially proposed by Sklar—is employed for dependency modeling. By Sklar’s theorem [24], for bivariate random variables X, Y, let $H\left(x,y\right)$ denote the joint distribution function with continuous marginal distributions $F\left(x\right)$ and G(y). Then, there exists a unique Copula function C(·):$I^{2}\to I=[0,1]$ such that

(12) $$H\left(x,y\right)=C\left(F\left(x\right),G\left(y\right)\right)$$

where C(F(x),G(y)) is the joint distribution function of variables X and Y, where $F\left(x\right)$ and G(y) are their marginal distributions.

Copula functions exhibit diverse families and parametric forms. Widely employed bivariate Copula functions in engineering reliability analysis include [25] the Archimedean Copula family, which, in turn, includes three widely applied types: Clayton Copula, Frank Copula, and Gumbel Copula. The elliptical Copula family comprises Gaussian Copula, Student’s t-Copula, and related variants, as specified in Table 1.

Research demonstrates significant differences among binary Copula functions in characterizing inter-variable dependencies and their dynamic sensitivity [26]. For mechanical systems such as wind turbine gearboxes, multi-scale failure correlations typically exhibit complex nonlinear characteristics that cannot be fully captured by a single Copula function [27]. The system’s failure correlation primarily stems from three mechanisms: Progressive degradation mechanisms (e.g., wear, pitting) cause sharply increased probabilities of associated failures when components reach advanced degradation states, characterized by intensified lower-tail dependence [28]. Random shock mechanisms (e.g., extreme loads) frequently induce instantaneous concurrent failures of multiple components, exhibiting upper-tail dependence [29]. Furthermore, non-extreme hybrid failure mechanisms intensify dependence within the median region of the distribution [30]. To accurately characterize these dependence properties, the Clayton Copula excels at capturing lower-tail dependence to model cumulative degradation effects, while the Gumbel Copula effectively describes upper-tail dependence representing shock-induced cascading failures, and the Frank Copula flexibly characterizes symmetrical median dependence. Consequently, the mixed Gumbel–Clayton–Frank Copula model comprehensively captures the system’s actual failure dependence structure by integrating these three functions. Based on the structural and operational characteristics of wind turbine gearbox systems, this study employs the mixed Copula functions. A nested copula architecture is designed for the reliability modeling of multi-scale failure dependencies, with the hybrid cumulative distribution function expressed as

(13) $$C\left(u,v\right)=\varphi_{1}C_{G}\left(u,v,\theta \right)+\varphi_{2}C_{C}\left(u,v,\alpha \right)+\varphi_{3}C_{F}\left(u,v,\gamma \right)$$

where $C_{G}$, $C_{C}$, and $C_{F}$ are the Gumbel, Clayton, and Frank Copula functions, respectively, and θ, α, and γ are their dependency parameters, quantifying correlation strength between variables u and v. $\varphi_{1}$, $\varphi_{2}$, and $\varphi_{3}$ are convex combination weights, characterizing the interdependency structure among variables, with the constraint $\varphi_{1}+\varphi_{2}+\varphi_{3}=1$.

#### 2.3.2. Correlation Parameter Estimation

The correlation parameter quantifies the interdependency strength between coupled mechanical systems. The most widely employed statistical inference techniques for correlation parameter estimation encompass maximum likelihood estimation and the method of moments. This study employs maximum likelihood estimation, combining the genetic algorithm with the residual sum of squares (RSS) criterion to estimate parameters $\varphi_{1}$, $\varphi_{2}$, $\varphi_{3}$, θ, α, and γ. The optimal parameters are obtained by minimizing the RSS value.

(14) $$\left\{\begin{array}{c} \begin{array}{l} RSS=\sqrt{\frac{1}{n}\sum_{k=1}^{n}(F_{emp}\left[{F\left(g_{1}\right)}_{i}^{k},{F\left(g_{2}\right)}_{i}^{k}\right]-{C\left[{F\left(g_{1}\right)}_{i}^{k},{F\left(g_{2}\right)}_{i}^{k}\right])}^{2}}\\ s.t.\theta \in [1,\infty )\\ \;\;\alpha \in (0,\infty )\\ \;\;\gamma \neq 0\\ \;\;0\leq \varphi_{i}\leq 1,\sum_{i=1}^{3}\varphi_{i}=1,(i=1,2,3) \end{array} \end{array}\right.$$

where $F_{emp}\left[{F\left(g_{1}\right)}_{i}^{k},{F\left(g_{2}\right)}_{i}^{k}\right]$ is the Monte Carlo-simulated functional response of individual gear pairs. The joint empirical distribution function for the stochastic sequence $\left[{F\left(g_{1}\right)}_{i}^{k},{F\left(g_{2}\right)}_{i}^{k}\right]$ (k=1,2,⋯,10,000) is generated through 10,000-cycle sampling. $C\left[{F\left(g_{1}\right)}_{i}^{k},{F\left(g_{2}\right)}_{i}^{k}\right]$ is the mixed Copula functions, where subscript i identifies the i-th gear pair within the transmission system. The convergence criterion is set to a relative error ≤ 0.5%, with a maximum number of iterations set at 10,000.

#### 2.3.3. Reliability Modeling with Failure Correlation Under Shock–Degradation Coupling

Let the initial contact fatigue strength of the tooth surface be $\sigma_{H0}$ and the initial bending fatigue strength of the tooth root be $\sigma_{F0}$, as specified in Formula (15), where all input parameters are modeled as normally distributed variables [31]. Monte Carlo simulations are performed to statistically derive the initial strength distribution for each gear.

(15) $$\left\{\begin{array}{c} \sigma_{H0}=\sigma_{Hlim}Z_{N}Z_{V}Z_{L}Z_{R}Z_{W}Z_{X}\\ \sigma_{F0}=\sigma_{Flim}Y_{ST}Y_{NT}Y_{\delta relt}Y_{RrelT}Y_{X} \end{array}\right.$$

where $\sigma_{Hlim}$ and $\sigma_{Flim}$ are the contact fatigue limit stress and root bending fatigue limit stress, respectively; $Z_{N}$ is the contact strength life coefficient; $Z_{V}$ is the velocity coefficient; $Z_{L}$ is the lubricant coefficient; $Z_{R}$ is the roughness coefficient; $Z_{W}$ is the work hardening coefficient; $Z_{X}$ is the size coefficient; $Y_{ST}$ is the stress correction coefficient; $Y_{NT}$ is the life coefficient; $Y_{\delta relt}$ is the root fillet sensitivity coefficient; $Y_{RrelT}$ is the root surface condition coefficient; and $Y_{X}$ is the size coefficient.

Within the framework of stress–strength interference theory, distinct limit state functions are formulated to separately address tooth surface contact fatigue and tooth root bending fatigue mechanisms for individual gears in wind turbine gearbox systems:

(16) $$\left\{\begin{array}{c} g_{1}=\sigma_{H0}-S_{H}-\sigma_{H}\\ g_{2}=\sigma_{F0}-S_{F}-\sigma_{F} \end{array}\right.$$

where $S_{H}$ is the cumulative degradation of tooth surface contact strength under shock–degradation coupling; and $S_{F}$ is the cumulative degradation of tooth root bending strength under shock–degradation coupling. The Monte Carlo simulation method was employed to conduct probabilistic sampling on the limit state functions $g_{1}$ and $g_{2}$, generating stochastic sample sequences. This process established the univariate empirical distribution functions $P_{1}$ and $P_{2}$ for each variable, as well as the multivariate joint empirical distribution function $C\left(P_{1},P_{2}\right)$. The gear failure probability P was subsequently derived through statistical aggregation of critical threshold exceedances:

(17) $$P=P\left(g_{1}\leq 0\cup g_{2}\leq 0\right)=P_{1}+P_{2}-C\left(P_{1},P_{2}\right)$$

The system reliability is quantified as

(18) $$R_{i}=1-P=1-\left[P_{1}+P_{2}-C\left(P_{1},P_{2}\right)\right]$$

**Figure 4:**
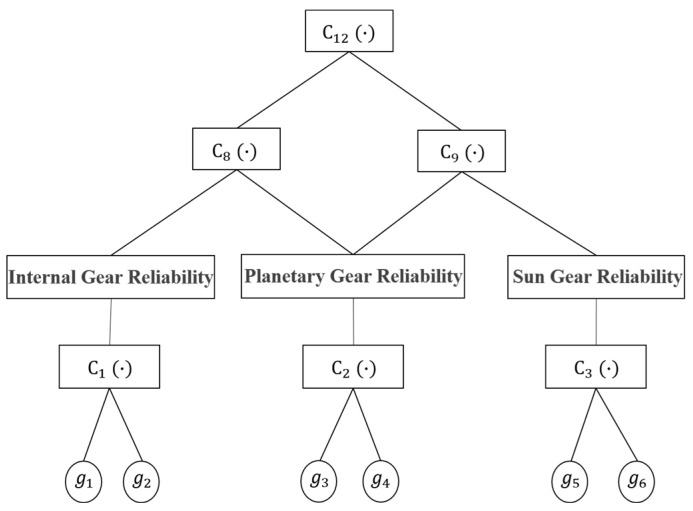
Schematic representation of the hierarchical nested Copula architecture for the planetary gear subsystem within a wind turbine gearbox.

Through systematic analysis of the operational principles and structural configuration of wind turbine gearbox systems, a fully nested architecture is implemented by constructing pairwise combinations of multi-failure modes and inter-component dependencies. This framework organizes binary copula functions into a multi-layered nested topology to holistically characterize system-level failure interdependencies, thereby enabling comprehensive reliability modeling of the gearbox system. Figure 4 illustrates the nested architectural topology of the planetary gear subsystem within the wind turbine gearbox, demonstrating the multi-layer dependency modeling framework integrating both component-level interactions and system-wide failure correlations.

In Figure 4, the nested Copula functions $C_{n}\left(\cdot \right),(n=1\sim 12)$ sequentially characterize two distinct correlation mechanisms from the base to apex layers: failure mode interdependencies and meshing gear pair interactions. Based on the hierarchical nested architecture illustrated in Figure 4 and integrated with Equation (19), the joint failure probabilities for each hierarchical level of the planetary gear stage are computed through progressive dependency aggregation:

(19) $$\left\{\begin{array}{c} P_{8}=P_{1}+P_{2}-C_{8}\left(P_{1},P_{2}\right)\\ P_{9}=P_{2}+P_{3}-C_{9}\left(P_{2},P_{3}\right)\\ P_{12}=P_{8}+P_{9}-C_{12}\left(P_{8},P_{9}\right) \end{array}\right.$$

where $P_{8}$ is the combined failure probability of the internal gear-planetary gear pair, $P_{9}$ quantifies the combined failure probability of the planetary gear-sun gear pair, and $P_{12}$ captures the system-level failure probability of the planetary gear stage.

In summary, the system reliability of the planetary gear stage within the wind turbine gearbox is quantified through progressive integration of multi-layer failure dependencies:

(20) $$R_{s}=R_{12}=1-P_{12}$$

## 3. Case Study and Numerical Verification

To validate the reliability model of wind turbine gearbox systems with failure correlation under shock–degradation coupling, this study investigates the planetary gear stage subsystem within a 2 MW wind turbine gearbox as the representative case. According to Reference [32], the design parameters of the wind turbine are as follows: The impeller has a radius of 28.8 m, a rated rotational speed of 14.8 r/min, and a rated torque of 1.27 × 10^6^ N·m. The wind conditions include an entry (cut-in) wind speed of 4 m/s, an exit (cut-out) wind speed of 20 m/s, a rated wind speed of 12 m/s, a wind density of 1.21 kg/m^3^, and a wind energy utilization coefficient of 0.55. The total transmission ratio of the system is 93. The internal gear is manufactured from 42CrMo steel, subjected to quenching and tempering followed by nitriding treatment, while the sun gear and planetary gears are made of 20CrMnTi steel with surface carburizing and quenching processes. The structural parameters of the planetary gear stage in the gearbox of this 2 MW wind turbine are detailed in Table 2.

Considering the actual operating conditions of gearbox systems in wind turbine units, the primary external excitation stems from natural wind, and its wind speed frequency distribution curve can be derived through mathematical curve fitting. Under normal conditions, the wind speed frequency distribution in a stable wind field follows the two-parameter Weibull distribution [33]. Hence, this study employs the two-parameter Weibull distribution curve to characterize the wind speed distribution pattern of the wind field. The mathematical representation of this relationship is formulated as

(21) $$f\left(v\right)={\frac{k}{c}\left(\frac{v}{c}\right)}^{k-1}\;exp\left[{-\left(\frac{v}{c}\right)}^{k}\right]$$

where $k={\left(\frac{\sqrt{D\left(v\right)}}{E\left(v\right)}\right)}^{-1.086}$ is the shape parameter of the Weibull distribution, $c=\frac{E\left(v\right)}{\Gamma \left(\frac{1}{k}+1\right)}$ is the scale parameter of the Weibull distribution, v denotes wind speed (m/s), E(v) represents the mean wind speed, and D(v) is the variance of wind speed.

**Figure 5:**
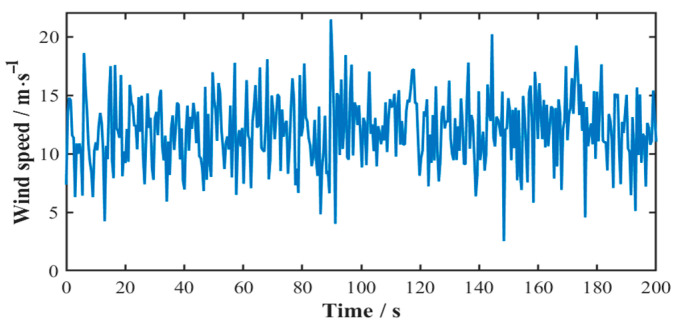
Time-history profile of random wind speed.

For a wind field characterized by a mean wind speed of 12 m/s and wind speed variance of 9 m^2^/s^2^, the Weibull distribution parameters are determined as shape parameter k = 4.5065 and scale parameter c = 13.1485. The synthesized 200 s time-history profile of the random wind speed model is presented in Figure 5.

**Figure 6:**
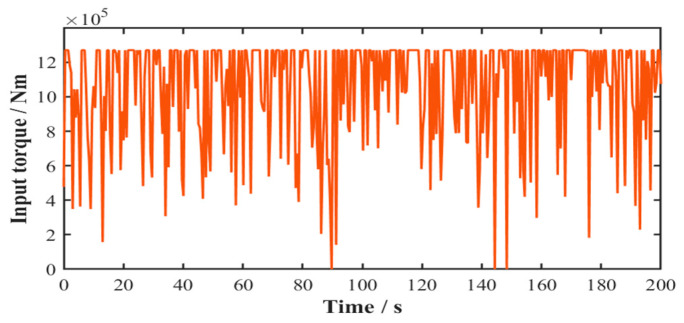
Time-history profile of system input torque.

Based on the structural parameters and operational principles of the wind turbine gearbox system, the relationship between the input torque $T_{in}$ and random wind speed V is established. The 200 s time-history profile of the gearbox input torque is presented in Figure 6.

(22) $$T_{in}=\left\{\begin{array}{ll} 0, & v<V_{cut\;in}\\ \frac{T_{rate}}{V_{rate}^{2}}\cdot v^{2}, & V_{cut\;in}\leq v<V_{rate}\\ T_{rate}, & V_{rate}\leq v\leq V_{cut\;off}\\ 0, & v>V_{cut\;off} \end{array}\right.$$

where $T_{in}$ denotes input torque (N·m), $T_{rate}$ represents rated torque (N·m), $V_{rate}$ indicates rated wind speed (m/s), v corresponds to random wind speed (m/s), $V_{cut\;in}$ specifies cut-in wind speed (m/s), and $V_{cut\;off}$ defines cut-out wind speed (m/s).

**Figure 7:**
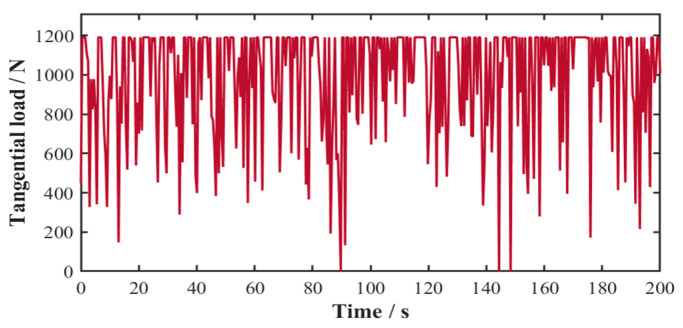
Time-history profile of tangential load in planetary gear stage system.

In accordance with ISO 6336-1 [34] for spur and helical gear load capacity calculations, the tangential gear loads were computationally determined throughout the gearbox. Using the structural parameters from Table 2, the 200 s time-history profile of tangential load variation in the planetary gear stage system is presented in Figure 7.

The contact stress on gear tooth surfaces and bending stress at gear tooth roots were analytically determined for each gear within the planetary gear stage system, with the computational methodologies detailed in Equations (23) and (24).

(23) $$\sigma_{H}=Z_{E}Z_{H}Z_{\varepsilon }Z_{\beta }\sqrt{\frac{K_{A}K_{V}K_{H\beta }K_{H\alpha }F_{t}}{d_{1}b}\cdot \frac{\left(q\pm 1\right)}{q}}$$

(24) $$\sigma_{F}=\frac{K_{A}K_{V}K_{F\beta }K_{F\alpha }F_{t}}{bm_{n}}Y_{F\alpha }Y_{S\alpha }Y_{\varepsilon }Y_{\beta }$$

where $Z_{E}$ is the elasticity coefficient, $Z_{H}$ is the zone coefficient, $Z_{\varepsilon }$ is the contact ratio coefficient, $Z_{\beta }$ is the helix angle coefficient, $K_{A}$ is the application coefficient, $K_{V}$ is the dynamic coefficient, $K_{H\beta }$ is the face load distribution coefficient, $K_{H\alpha }$ is the transverse load distribution coefficient, $F_{t}$ is the tangential load at reference circle, $d_{1}$ is the pinion reference diameter, b is the effective face width, q is the teeth number ratio, $K_{F\beta }$ is the face load distribution coefficient, $K_{F\alpha }$ is the transverse load distribution coefficient, $m_{n}$ is the normal module, $Y_{F\alpha }$ is the form coefficient at tooth tip loading, $Y_{S\alpha }$ is the stress correction coefficient at tooth tip loading, $Y_{\varepsilon }$ is the contact ratio coefficient, and $Y_{\beta }$ is the helix angle coefficient.

**Figure 8:**
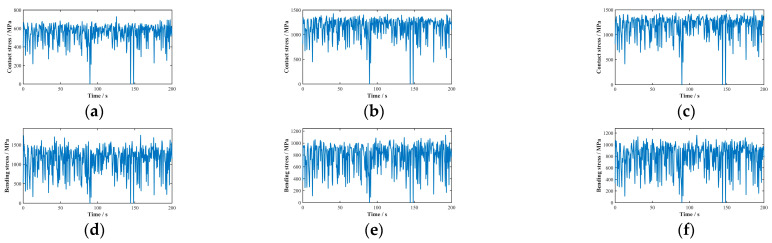
Stress distribution characteristics in planetary gear stage system: (**a**) internal gear contact stress; (**b**) planetary gear contact stress; (**c**) sun gear contact stress; (**d**) internal gear bending stress; (**e**) planetary gear bending stress; (**f**) sun gear bending stress.

The Monte Carlo random sampling method was used to solve Equations (23) and (24), using input parameter values from reference [32]. The resulting contact stress distribution curves on the tooth surfaces and root bending stress distribution curves of each gear in the planetary gear system are shown in Figure 8.

**Figure 9:**
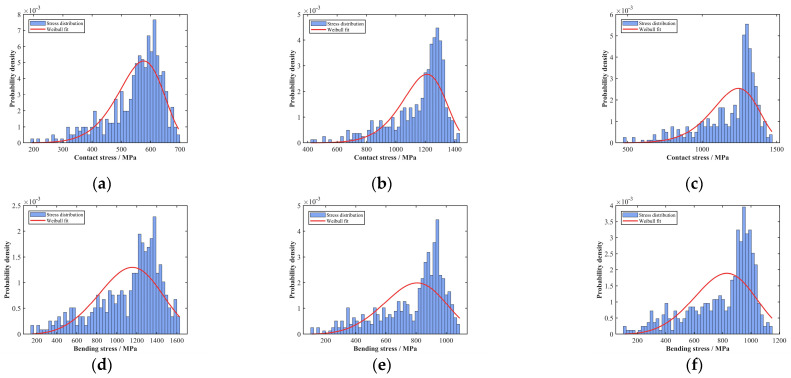
Stress distribution fitting curves in the planetary gear stage system: (**a**) internal gear contact stress; (**b**) planetary gear contact stress; (**c**) sun gear contact stress; (**d**) internal gear bending stress; (**e**) planetary gear bending stress; (**f**) sun gear bending stress.

Fitting analysis of stress values for each gear in the planetary gear system determines the probability density fitting curves, as depicted in Figure 9.

In mechanical system reliability analysis, the Weibull distribution is extensively employed for probabilistic characterization of component failures. Through the parametric adaptation of its characteristic shape and scale factors, this distribution accurately characterizes the stress profile evolution in gear transmission systems. Analysis of the probability density fitting curves for stress values across all gears in the aforementioned planetary gear-stage system reveals convex profiles with a distinct peak (single local maximum). Consequently, both the contact stress on gear tooth surfaces and bending stress at gear roots within the system are optimally characterized by the two-parameter Weibull distribution. The derived Weibull distribution parameters characterizing stress profiles are summarized in Table 3.

Based on the parameter estimation methodology for the wind turbine gearbox system illustrated in Figure 3, the degradation parameters of all gears in the planetary gear-stage system are calculated and tabulated in Table 4.

**Figure 10:**
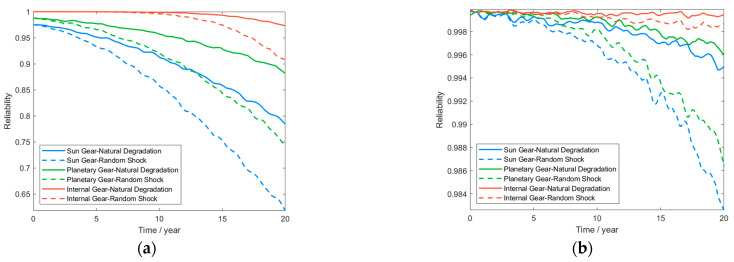
Time-dependent reliability profiles of the planetary gear-stage system under dual failure modes: (**a**) contact fatigue reliability; (**b**) bending fatigue reliability.

Based on the actual service conditions of wind turbine gearbox systems, the random impact parameters are defined as follows: arrival rate λ = $5\times 10^{-5}h^{-1}$, impact intensity $W_{i}\sim N\left(400,\;40^{2}\right)\;MPa$, and conversion factor $k=2\times 10^{-2}$ [35]. Using Equation (11), the 20-year contact fatigue reliability and bending fatigue reliability were computed for all gear components in the wind turbine gearbox’s planetary gear-stage system. The time-dependent reliability profiles of each gear component are presented in Figure 10.

Figure 10 demonstrates that under contact fatigue failure conditions in the planetary gear-stage system, the reliability of all gear components exhibits nonlinear degradation from an initial value of 0.99 to below 0.65 with operational duration. Under root bending fatigue failure conditions, all gear components maintain reliability above 0.98 yet exhibit a decreasing trend over time. This observation aligns with the operational reliability degradation patterns observed in wind turbine gearbox systems under actual service conditions over time.

Figure 10a demonstrates that under natural degradation conditions, gear reliability decreases gradually in an approximately linear manner, attributable to progressive surface wear mechanisms (microscopic plastic deformation, crack initiation, and propagation). The degradation rate depends on the material’s wear resistance. During actual gearbox operation, load fluctuations and variations in lubrication conditions can disrupt this linear degradation accumulation pattern. The introduction of random shocks accelerates reliability degradation. This occurs because shocks not only instantaneously amplify contact stress—inducing overload damage (e.g., cracks, plastic deformation)—but also generate residual damage (e.g., residual stress, surface indentations/scratches). These defects subsequently act as stress concentration sources during natural degradation processes. During actual gearbox operation, dynamic coupling effects prove critically significant: System vibrations initiated by shocks disrupt gear meshing conditions and lubrication (oil film rupture), thereby forming a vicious cycle: shock → vibration → lubrication deterioration → stress amplification. Concurrently, transient deformations of components (e.g., bearings, shafts) instantaneously redistribute loads, indirectly altering force distribution patterns. Comparative analysis of contact fatigue reliability curves for tooth surfaces across gear types shows that the sun gear exhibits the most severe reliability degradation owing to elevated dynamic stresses generated by multi-point shock loading at its central engagement position. Planetary gears achieve enhanced reliability through load-sharing that mitigates shock effects. However, actual load distribution effectiveness is compromised by manufacturing tolerances and component stiffness variations. The internal gear effectively mitigates localized stress fluctuations on tooth surfaces due to its fixed support rigidity and mass. Under both operating regimes, the risk of contact fatigue failure approaches zero. Conversely, this transfers large-magnitude shock reaction forces to structural components (e.g., gearbox housing), demonstrating dynamic coupling transfer of failure modes.

Figure 10b demonstrates that under root bending fatigue failure conditions with solely natural material degradation considered, all gears exhibit gradual, minor reliability degradation over time. This trend aligns with the progressive crack propagation failure mechanism. Random shock loads significantly accelerate fatigue crack initiation and propagation. This failure mechanism arises primarily through sudden contact stress perturbation and instantaneous overload at the tooth root during transient loading events. In the comparative analysis of root bending fatigue reliability curves across gear types, the sun gear exhibits the most severe reliability degradation under shock loading. This failure mechanism involves both inherent stress concentration characteristics at its central position and shock-induced dynamic amplification superimposed on cyclic stresses, accelerating cumulative damage kinetics. Planetary gears leverage multi-planet load sharing to disperse shock forces, demonstrating superior shock resistance. The internal gear exhibits the lowest failure probability owing to its high structural rigidity, which effectively mitigates dynamic bending stress peaks and limits deformation. These phenomena fundamentally reveal that during actual gearbox operation, complex dynamic coupling effects exist among failure modes and mechanisms—including natural degradation, shock disturbances, and structural responses—which collectively govern the system’s reliability evolution.

Based on the characteristics of wind turbine gearbox systems, the multi-scale correlation parameters were calculated using the previously defined RSS methodology through Equation (14), with results presented in Table 5.

**Figure 11:**
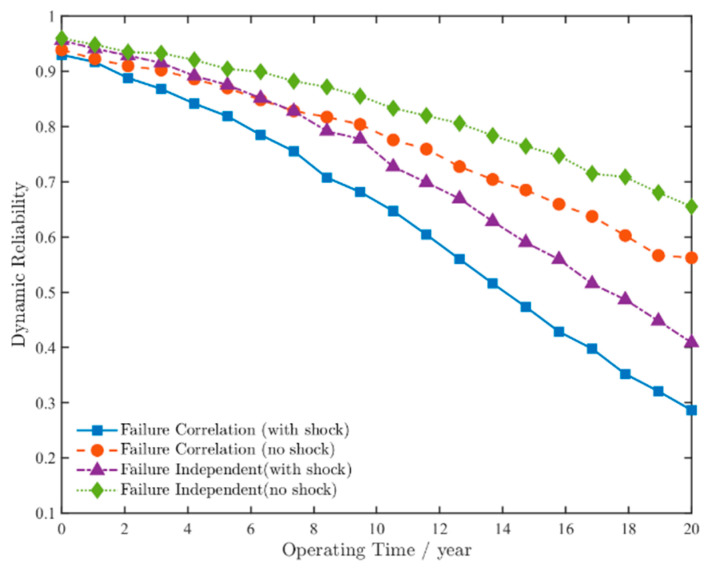
Dynamic reliability curves of the planetary gear-stage system.

Based on the preceding analysis of the planetary gear-stage system, the contact fatigue failure probabilities, root bending fatigue failure probabilities, and corresponding correlation parameters for all gears were substituted into Equations (19) and (20). The dynamic reliability was calculated under three analytical frameworks: (1) assuming mutual independence among gears, (2) considering only natural degradation effects, and (3) accounting for failure correlation under shock–degradation coupling. The time-dependent reliability curves for these scenarios are presented in Figure 11.

As shown in Figure 11, the reliability of the planetary gear-stage system gradually decreases over time, with all four curves exhibiting consistent downward trends. This validates the effectiveness of the model presented in this paper.

When considering system failure correlation, after 10 years of operation, the system’s reliability decreases to approximately 0.78 under natural degradation alone. When accounting for random shock loads, reliability further declines to around 0.65. This demonstrates that random shock events accelerate system degradation, consistent with previous analyses of shock-induced stress amplification and cumulative damage mechanisms. Under shock–degradation coupling conditions, the system reliability drops below 0.85 after nearly 6 years of operation under failure-independent reliability analysis. In contrast, when considering system failure correlation, the reliability falls below 0.85 after approximately 4 years of operation. This aligns with the actual operating conditions of wind turbine gearbox systems: During normal operation, the failure of one gear accelerates the failure process of another, and there exists a positive correlation between failures of interconnected gears. Meanwhile, contact fatigue failure on gear tooth surfaces induces meshing shocks, increases the stress amplitude at tooth roots, and, thereby, initiates or propagates bending cracks. Bending fatigue failure at the tooth root alters the meshing position, induces eccentric loading or edge contact, and accelerates contact fatigue failure on the tooth surface. Therefore, the rationality of the model established in this study has been validated.

Figure 11 demonstrates that the reliability profiles across all four scenarios exhibit consistent degradation trends. Regardless of whether random shock loads are considered, accounting for failure correlation results in faster reliability degradation compared to the assumption of independent reliability values. Considering only the degradation of the system performance itself, the failure rate has increased by approximately 40%. When considering the shock–degradation coupling, the failure rate increased by approximately 37%. The failure rate of the method proposed in this study has increased by over 60% compared to considering natural degradation alone. This demonstrates that the model established through the methodology proposed in this study more accurately reflects the actual operating conditions of wind turbine gearbox systems. It not only validates the model’s validity and rationality but also provides a theoretical foundation for reliability design, life prediction, and reliability enhancement in wind turbine gearbox system engineering.

## 4. Conclusions

In this study, a dynamic reliability model for wind turbine gearbox systems under shock–degradation coupling is developed, grounded in the actual operational conditions of these systems. The validity and rationality of the model are verified and analyzed using the planetary gear-stage system from a 2 MW wind turbine gearbox as a case study. This study comprehensively considers the influence of random shock loads on wind turbine gearbox systems. By integrating analyses across failure mode, component, and system levels, a reliability model incorporating failure correlations under shock–degradation coupling is established using mixed Copula functions. The planetary stage of a 2 MW wind turbine gearbox was analyzed under four dynamic reliability scenarios: natural degradation only, shock–degradation coupling, natural degradation with failure dependence, and shock–degradation coupling with failure dependence. The proposed model aligns with established wind turbine gearbox degradation frameworks while incorporating critical enhancements. In this study, the consideration of random shock loads and varying failure correlations in the gearbox system serves not only to address the significant amplification effects of shock loads on gear contact stresses and the differences in failure correlation levels, but also to enhance the model’s fidelity in replicating real-world operational conditions of wind turbine gearbox systems. This ensures the developed model is both more rational and effective in practical applications.

Through comparative analysis of the four methodologies, the modeling approach proposed in this study exhibits comparatively lower reliability, corresponding to a higher failure rate. In this way, not only are the desired results more rational, but the design, manufacturing, and maintenance costs of wind turbine systems are also reduced, thereby improving economic efficiency. However, the modeling methodology for wind turbine gearbox systems explored in this study remains confined to the planetary gear-stage system and considers only wind speed as an environmental input. For the actual gearbox in wind turbine units, the encountered wind farm environment is highly complex. However, the current model simplifies the load process (utilizing the HPP assumption) and Copula time-varying behavior; furthermore, it focuses solely on the planetary gear subsystem. It is extremely difficult to accurately characterize failure logic using only wind farm wind speed. In practical engineering applications, specific calculations and derivations must be performed based on the actual conditions. Future work should develop non-stationary load models and a time-varying Copula framework, extending these to the entire drivetrain system and digital twin applications.

## Figures and Tables

**Table 1:** Widely used bivariate Archimedean Copula functions.

Function Type	$C(u_{1},u_{2},\theta )$	Parameter Range
Gumbel Copula	$exp\{-[(-lnu_{1}{)}^{\theta }+(-lnu_{2}{)}^{\theta }{]}^{\frac{1}{\theta }}\}$	[1,∞)
Clayton Copula	$(u_{1}^{-\theta }+u_{2}^{-\theta }-1{)}^{-\frac{1}{\theta }}$	(0,∞)
Frank Copula	$-\frac{1}{\theta }ln[1+\frac{\left(e^{-\theta u_{1}}-1)(e^{-\theta u_{2}}-1\right)}{e^{-\theta }-1}]$	(−∞,0)∪(0,+∞)

**Table 2:** Structural parameters of planetary gear stages in the 2 MW wind turbine gearbox [32].

Structure	Gear Type	Number of Teeth	Module	Pressure Angle	Helix Angle
Planetary Gear stage	Sun Gear	27	16	23°	0
	Planetary Gear	44	16	23°	0
	Internal Gear	115	16	23°	0

**Table 3:** Weibull distribution parameters for stress characterization in the planetary gear-stage system.

Gear Type	Contact Stress		Bending Stress	
	Shape Parameter k	Scale Parameter c	Shape Parameter k	Scale Parameter c
Internal Gear	586.8736	8.1927	1227.1098	4.2807
Planetary Gear	1226.8694	8.7388	846.8270	4.5636
Sun Gear	1260.7116	8.6134	877.3425	4.3766

**Table 4:** Degradation parameters of gears in the planetary gear-stage system.

Gear Type	Contact Fatigue Strength Degradation		Bending Fatigue Strength Degradation	
	*α*(t)	*β*	*α*(t)	*β*
Internal Gear	0.0004t	1.5712	0.0003t	0.6386
Planetary Gear	0.0011t	0.6142	0.0010t	0.2695
Sun Gear	0.0011t	0.6658	0.0011t	0.3055

**Table 5:** Correlation parameters of the planetary gear-stage system.

Copula Function	$\varphi_{1}$	$\varphi_{2}$	$\varphi_{3}$	θ	α	γ
$C_{1}\left(\cdot \right)$	0.0053	0.0627	0.9320	1.0418	0.0052	0.0459
$C_{2}\left(\cdot \right)$	0.0163	0.2813	0.7024	1.0381	0.0157	0.0525
$C_{3}\left(\cdot \right)$	0.0615	0.4468	0.4917	1.0187	0.0503	0.1618
$C_{8}\left(\cdot \right)$	0.3300	0.3300	0.3400	1.0400	0.0100	0.0500
$C_{9}\left(\cdot \right)$	0.3300	0.3300	0.3400	1.0400	0.0400	0.0100
$C_{12}\left(\cdot \right)$	0.3000	0.3000	0.4000	1.0500	0.0200	0.0300

## Data Availability

Data are contained within the article.
